# Draft genome of a clinical *Pseudomonas qingdaonensis *isolate from a hospitalized patient at Kenyatta National Hospital, Kenya

**DOI:** 10.12688/f1000research.127935.1

**Published:** 2022-12-21

**Authors:** Brian Ogoti, Racheal Gachogo, Frank Onyambu

**Affiliations:** 1 Centre for Epidemiological Modelling and Analysis, University of Nairobi, Nairobi, Kenya; 2 Institute of Tropical and Infectious Diseases, University of Nairobi, Nairobi, Kenya; 3 Division of Immunology, Department of Human Pathology, University of Cape Town, Cape Town, South Africa; 4 Meru University of Science and Technology, Meru, Kenya; 5 Centre for Molecular Biosciences and Genomics, Nairobi, Kenya

**Keywords:** Draft genome, Pseudomonas qingdaonensis

## Abstract

The first draft genome of *Pseudomonas qingdaonensis*, a gram-negative bacteria isolated from hospitalized patients in Kenya, is presented in this study. The genome was assembled using nanopore MinION readings, yielding an assembly of 8,857,650 base pairs made up of 5(five) contigs. The genome sequence of *Pseudomonas qingdaonensis* will help researchers better grasp its genetics. Furthermore, the data can be a valuable resource for comparative genomics and future research in this novel species.

## Abbreviations

MLST: Multilocus sequence typing

rRNA: Ribosomal RNA

tRNA: Transfer RNA

## Introduction

The diverse Gram-negative genus *Pseudomonas* contains species isolated from varied habitats, plants, animals, and people. It is a gram negative, aerobic, rod-shaped belonging to the gammaproteobacteria. ^1^ Here we describe a novel species *Pseudomonas qingdaonensis* isolated from a hospitalized patient in Kenya. Its genome sequencing will aid in our understanding of this pathogen's biology. The draft genome assembled here will enable more in-depth studies to be conducted in specific genome sections or genes that promote the novel *Pseudomonas* species reported here in the colonization of hospitalized patients.

## Methods

### Sample collection

The *Pseudomonas qingdaonensis* was isolated from a hospitalized patient at Kenyatta National hospital in Kenya. A stool sample was collected from the hospitalized patient and in the laboratory, it was cultured on MacConkey agar (HiMedia Lab, Mumbai, India) at 37°C for 18 hours. The colonial morphology on MacConkey agar plate, catalase, oxidase and gram staining procedure tests were used in identification. ^2^ The colonies on MacConkey were flat, 2-3mm, smooth colonies with an irregular leafy margin. Under the microscope after gram staining procedure, gram negative rods measuring 0.6-0.8 μm were identified. On the biochemical tests, the colonies isolated were catalase positive, and oxidase positive. Confirmation of the ID of *Pseudomonas species* was done using the VITEK ^®^ 2 (Biomerieux, France). Before loading isolates into the VITEK ^®^ 2, bacterial suspensions were done by emulsifying the isolates in 0.5% saline and standardizing turbidity to 0.5 McFarland’s using a densitometer. The suspension was used for species ID and AST in the VITEK ^®^ 2 using Gram-negative ID cards (ID-GN 21341) and results analyzed according to Clinical and Laboratory Standards Institute guidelines. ^3^

### DNA extraction and library preparation

A single colony was sub-cultured on nutrient agar (HiMedia Lab, Mumbai, India) at 37°C for 18 hours. Colonies from nutrient agar were resuspended in 400 ul of phosphate buffered saline and then extracted using ISOLATE II Genomic DNA Kit (Meridian Bioscience Inc, London, UK). The DNA was eluted with pre-warmed nuclease free water in a total volume of 50 ul. Sequencing library preparation was done using the Oxford Nanopore genomic sequencing kit SQK-LSK109 (ONT, UK) and Native Barcoding expansion kits (EXP-NBD104, EXP-NBD114) as per the manufacturer’s instructions. Sequencing was performed using the MinKNOW software (ONT, United Kingdom, Oxford) with live Base calling turned on. The quality score of the FAST5 and subsequent FASTQ files produced by MinKNOW was set at 7.

### Bio-informatics analyses

Live base calling and adapter trimming was performed using Guppy v3.6.1 ( https://nanoporetech.com). De-novo assembly of the *Pseudomonas qingdaonensis* was performed using UNICYCLER v0.4.9 ( https://github.com/rrwick/Unicycler). ^4^ The genome quality and assembly was contrasted and carefully selected based on the following criteria: the size of contigs, GC (%) content, N50 length, and genome coverage as determined by QUAST v5.2.0 ( http://bioinf.spbau.ru/quast). Visualization of the draft assembly was done using Bandage v0.9.0 ( https://rrwick.github.io/Bandage). ^5^ Genome annotation of the draft assembly was done using the NCBI Prokaryotic Genome annotation pipeline (PGAP) ( https://www.ncbi.nlm.nih.gov/genome/annotation_prok/).

### Ethics declarations

The Kenyatta National Hospital – University of Nairobi (KNH-UON) Ethics and Research Committee was sought for approval and approved under Ref no. P391/07/2020. Signed informed consent, approved under Ref no. P391/07/2020, was obtained from the participant and the project followed ethical principles and guidelines for research involving human subjects.

## Results

The total length of the *Pseudomonas qingdaonensis* was 8,857,650 base pairs long consisting of 5(five) contigs. The N50 contig was 3332773 and the GC% content was 64.03% ( Table 1). The total coverage of the draft genome was 51X.

**Table 1.** QUAST statistics of the *Pseudomonas qingdaonensis* draft genome.

Assembly	Assembly
# contigs (≥ 0 bp)	5
# contigs (≥ 1000 bp)	5
# contigs (≥ 5000 bp)	5
# contigs (≥ 10000 bp)	4
# contigs (≥ 25000 bp)	4
# contigs (≥ 50000 bp)	4
Total length (≥ 0 bp)	5857650
Total length (≥ 1000 bp)	5857650
Total length (≥ 5000 bp)	5857650
Total length (≥ 10000 bp)	5851293
Total length (≥ 25000 bp)	5851293
Total length (≥ 50000 bp)	5851293
Number of contigs	5
Largest contig	3332773
Total length	5857650
GC (%)	64.03
N50	3332773
N90	889928
L50	1
L90	3

#- Number of.

The draft genome was submitted to NCBI GenBank repository under the accession number JANWGM000000000.1. The genome annotation done using PGAP showed the presence 6,274 genes with 3,090 being coding genes, 19 rRNAs and 70 tRNAs ( Table 2).

**Table 2.** PGAP annotation results of the *Pseudomonas qingdaonensis* genome.

Genes (total)	6,274
CDSs (total)	6,179
Genes (coding)	3,090
CDSs (with protein)	3,090
Genes (RNA)	95
rRNAs	7, 6, 6 (5S, 16S, 23S)
complete rRNAs	7, 6, 6 (5S, 16S, 23S)
tRNAs	70
ncRNAs	6
Pseudo Genes (total)	3,089
CDSs (without protein)	3,089
Pseudo Genes (ambiguous residues)	0 of 3,089
Pseudo Genes (frameshifted)	2,865 of 3,089
Pseudo Genes (incomplete)	398 of 3,089
Pseudo Genes (internal stop)	63 of 3,089
Pseudo Genes (multiple problems)	228 of 3,089

The draft sequence of *Pseudomonas qingdaonensis* was submitted to the PubMLST website for multilocus sequence typing ( https://pubmlst.org/) to generate an in-silico MLST profile. Table 3 show the allelic matches from PUBMLST.

**Table 3.** PubMLST sequence query of *Pseudomonas qingdaonensis.*

Locus	Allele	Length	Contig	Start position	End position
BACT000004 (rpsD)	9850	621	1	1883670	1884290
BACT000005 (rpsE)	16706	501	1	1880153	1880653
BACT000006 (rpsF)	7506	426	1	671121	671546
BACT000008 (rpsH)	8099	393	1	1878852	1879244
BACT000009 (rpsI)	9124	393	1	2359896	2360288
BACT000010 (rpsJ)	7797	312	1	1872060	1872371
BACT000011 (rpsK)	7715	390	1	1883265	1883654
BACT000013 (rpsM)	7758	357	1	1882878	1883234
BACT000015 (rpsO)	7632	270	1	2165238	2165507
BACT000016 (rpsP)	7203	252	4	42015	42266
BACT000017 (rpsQ)	6904	267	1	1876787	1877053
BACT000018 (rpsR)	6844	231	1	670863	671093
BACT000021 (rpsU)	5073	216	1	1798529	1798744
BACT000033 (rplD)	9563	603	1	1873100	1873702
BACT000042 (rplM)	8392	429	1	2359453	2359881
BACT000043 (rplN)	7772	369	1	1877077	1877445
BACT000045 (rplP)	8019	414	1	1876180	1876593
BACT000046 (rplQ)	7980	387	1	1885355	1885741
BACT000047 (rplR)	7415	351	1	1879799	1880149
BACT000050 (rplU)	7363	312	1	374142	374453
BACT000051 (rplV)	7664	333	1	1875136	1875468
BACT000052 (rplW)	7143	300	1	1873699	1873998
BACT000057 (rpmB)	6464	237	1	1376589	1376825
BACT000058 (rpmC)	5619	192	1	1876593	1876784
BACT000059 (rpmD)	5467	177	1	1880656	1880832
BACT000060 (rpmE)	7515	216	1	1639401	1639616
BACT000061 (rpmF)	6317	183	1	2965257	2965439
BACT000062 (rpmG)	3770	156	1	1376837	1376992
BACT000063 (rpmH)	5737	135	1	1251295	1251429
BACT000064 (rpmI)	6315	195	1	3274217	3274411
BACT000065 (rpmJ)	5114	117	1	1882630	1882746

## Limitations

Comparative analyses were not undertaken, and more research is needed to identify *Pseudomonas qingdaonensis* relationship to other *Pseudomonas qingdaonensis* isolates globally.

## Data Availability

GenBank: Pseudomonas qingdaonensis, whole genome sequence, Accession number JANWGM000000000.1: https://www.ncbi.nlm.nih.gov/Traces/wgs/JANWGM01?display=contigs BioProject: *Pseudomonas qingdaonensis strain: CMB_001*, Accession number PRJNA869907: https://identifiers.org/NCBI/bioproject:PRJNA869907 BioSample: Pathogen: clinical or host-associated sample from Pseudomonas qingdaonensis, Accession number SAMN30333618: https://identifiers.org/biosample:SAMN30333618
