# Supplementary material for: Draft genome of a clinical Pseudomonas qingdaonensis isolate from a hospitalized patient at Kenyatta National Hospital, Kenya
Source: F1000Res. 2022 Dec 21;11:1544. [Version 1] doi: 10.12688/f1000research.127935.1 (PMC10154912; doi:10.12688/f1000research.127935.1)
Supplement: Peer review history

# Peer review: Reviewer response for version 1

**DOI:** 10.5256/f1000research.140481.r168571

**Published:** 2023-05-02

**Authors:** Aquillah Kanzi

**Affiliations:** 1 Department of Biochemistry, Genetics and Microbiology, Forestry and Agricultural Biotechnology Institute, University of Pretoria, Pretoria, Gauteng, South Africa

The authors sequenced and assembled a draft genome of *Pseudomonas qingdaonensis *isolated from a hospitalised patient. The methodology used for sequencing and assembly is technically sound. The comments and suggestions below can improve the quality of this work.

**Introduction**

 1. The authors use of "novel species" in the introduction is misleading.

**Bio-informatics analyses**

 2. The authors used QUAST to assess the quality of the assembled genome. Was a reference genome used? e.g. GCF_021601925.1

**Results**

 3. The stated size of the draft genome in the results section 8,857,650 differs from the QUAST statistics in table 1.

 4. There is no visual presentation of the draft genome. Visualisation was described in the Bio-informatics analyses section but no genome map was shown. A visual presentation will improve the quality of this work

 5. The results shown in Table 3 are not comprehensible. Yes, there are matching MLST profiles in PUBMLST but what does this mean?

 The article needs a short discussion section to put into context the main findings.

Are the datasets clearly presented in a usable and accessible format, and the assembly and annotation available in an appropriate subject-specific repository?

Yes

Are sufficient details of the sequencing and extraction, software used, and materials provided to allow replication by others?

Yes

Are the rationale for sequencing the genome and the species significance clearly described?

Yes

Are the protocols appropriate and is the work technically sound?

Yes

Reviewer Expertise:

Genomics, structural bioinformatics, genetics and microbiology

I confirm that I have read this submission and believe that I have an appropriate level of expertise to confirm that it is of an acceptable scientific standard.

# Peer review: Reviewer response for version 1

**DOI:** 10.5256/f1000research.140481.r158492

**Published:** 2023-01-03

**Authors:** Gerald Mboowa

**Affiliations:** 1 Department of Immunology and Molecular Biology, School of Biomedical Sciences, Makerere University College of Health Sciences, Kampala, Uganda

Take note that this is written as Gram-negative not gram-negative throughout the entire write up.

 Authors should include the following to make this work more informative:

1. Where was sequencing done?
2. Annotation of antimicrobial resistance genes and virulence factors should be included
3. Clinical history and demographics of the patient

Include a brief discussion of the findings from this genome assembly.

Are the datasets clearly presented in a usable and accessible format, and the assembly and annotation available in an appropriate subject-specific repository?

Yes

Are sufficient details of the sequencing and extraction, software used, and materials provided to allow replication by others?

Yes

Are the rationale for sequencing the genome and the species significance clearly described?

Yes

Are the protocols appropriate and is the work technically sound?

Yes

Reviewer Expertise:

Bioinformatics & Genomics

I confirm that I have read this submission and believe that I have an appropriate level of expertise to confirm that it is of an acceptable scientific standard.
